# Prediction of aggregation in monoclonal antibodies from molecular surface curvature

**DOI:** 10.1038/s41598-025-13527-w

**Published:** 2025-08-02

**Authors:** Benjamin Knez, Lara Erzin, Žiga Kos, Drago Kuzman, Miha Ravnik

**Affiliations:** 1 Novartis LLC, Verovškova 57, 1000 Ljubljana, Slovenia; 2 Faculty of Mathematics and Physics, University of Ljubljana, Jadranska 19, 1000 Ljubljana, Slovenia; 3 International Institute for Sustainability with Knotted Chiral Meta Matter (WPI-SKCM2), Higashi-Hiroshima, Japan; 4 Department of Condensed Matter Physics, Jožef Stefan Institute, Ljubljana, Slovenia

**Keywords:** Biomaterials - proteins, Biomedical materials, Soft materials

## Abstract

Protein aggregation is one of the key challenges in the biopharmaceutical industry as its control is crucial in achieving long-term stability and efficacy of biopharmaceuticals. Attempts have been made to develop regression models for predicting the aggregation of monoclonal antibodies in solution using machine learning methods. These efforts have yielded varying levels of success, with current state-of-the-art AI approaches achieving good prediction accuracies ($r=0.86$). Here, we demonstrate the prediction of aggregation rate in monoclonal antibodies with beyond state-of-the-art reliability using a coupled AI-MD-Molecular surface curvature modelling platform. The scientific novelty of this approach lies in using local geometrical surface curvature of proteins as the core element for protein stability analysis. By combining local surface curvature and hydrophobicity, as derived from time-dependent MD simulations, we are able to construct aggregation predictive features that, when coupled with linear regression machine learning techniques, give a high prediction accuracy ($r=0.91$) on a dataset of 20 molecules. More generally, this approach shows significant potential for quantitative in silico screening and prediction of protein aggregation, which is of great scientific and industrial relevance, particularly in biopharmaceutics.

## Introduction

Protein aggregation is crucial in the development of biotherapeutic formulations, as it affects the stability and safety of biological drugs^1–4^. Especially, understanding protein aggregation at the structural level is vital for selecting the right molecular candidates and for formulating safe and stable biological drugs^5^. Moreover, protein aggregation also plays a pivotal role in designing drug delivery systems that prioritize the needs of patients. Finally, the biopharmaceutical industry’s shift towards high-concentration antibody formulations for subcutaneous administration also requires strong control and understanding of protein aggregation^6,7^.

The protein’s three-dimensional structure is linked to its function. Experimental methods such as X-ray crystallography, NMR and cryo-electron microscopy are today strong approaches for obtaining protein structures^8,9^. In parallel, computational methods for protein structure prediction are being developed with increasing accuracy and are emerging as possible fast and cost effective alternatives^10–12^. Major current computational tools include AlphaFold^13^, RoseTTAFold^14^, OmegaFold^15^ and ESMFold^16^. Due to the structural fluctuations that naturally occur in proteins, and are commonly responsible for their diverse biological functionality, a single calculated structure of a protein is often insufficient for their understanding. Molecular dynamics simulations offer a profound complementary tool to the AI methodologies^17,18^ as they enable the study of the complex motion of proteins at the atomic level over time and according to exact dynamic equations^19,20^. This dynamic perspective allows studying phenomena such as protein folding^21^, protein-ligand binding^22^, thermal stability of antibodies^23^ and more.

An emerging strategy for studying the degradation of biopharmaceuticals involves describing therapeutic protein properties using designated molecular descriptors and surface features^24–28^ which reduce the dimensionality of structural and physico-chemical variables while retaining essential information. Descriptors can be sequence-based, such as the frequency of distinct motifs of individual amino acids in the chain^29^, amino-acid charge, secondary structure, etc.^30^. However, these sequence-based descriptors do not capture various relevant structural-based mechanisms of protein aggregation and thus structure-based molecular descriptors are better suited for predicting aggregation^31,32^. For example, the solvent-accessible surface area (SASA) is an indicator of interaction surface and spatial aggregation propensity (SAP^25^) identifies aggregation-prone regions. SAP is often combined with net charge into the Developability Index (DI)^26^, which is a composite metric that considers solubility to predict aggregation. The spatial charge map (SCM^24^) is based on the partial charge of solvent-exposed atoms and accounts for electrostatic interactions that underlie viscosity. It has been utilized to accurately rank high-concentration mAb solution viscosities across various industrial pipelines^24,33^. Another descriptor uses fine-grained points and patches on the solvent accessible surface, on which physico-chemical properties are then projected and optimized against a test dataset^34^. Finally, recent attempts use multiple descriptors as coupled via machine learning algorithms to predict the aggregation rates of high-concentration mAb solutions^35,36^.

Here, we develop a predictive experimentally-informed AI and MD modelling platform for aggregation of monoclonal antibodies in biopharmaceutical formulations, of direct relevance for fundamental science and industrial applicability. Specifically, the prediction platform rather uniquely combines a coupled series of state-of-the-art methodologies (AlphaFold $\rightarrow$ Molecular dynamics $\rightarrow$ Protein features calculation $\rightarrow$ Experimentally informed ML) to predict the aggregation rate of mAbs from the amino-acid sequence. The platform is validated on publicly available experimental data of 20 mAb proteins, obtaining excellent prediction accuracy with predicted to experimental correlation coefficient of $r=0.91$. The core scientific novelty is the recognition of local protein surface curvature, introduced as a special geometrical surface feature (molecular descriptor), which in combination with hydrophobicity we show strongly correlates with the protein aggregation rate. Finally, this work is discussed in the context of its applicability in the biopharmaceutical industry.

## Results

**Fig. 1:**
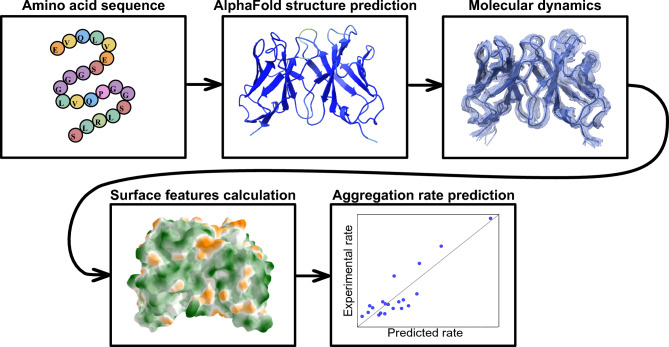
AI-MD-Molecular surface curvature modelling platform for monoclonal antibody aggregation rate prediction. Using the amino acid sequence, we construct a 3D antibody structure, employ it in a molecular dynamics simulation, calculate surface features for all frames, and then use this data to build a machine learning model for predicting antibody aggregation rates.

The prediction of monoclonal antibody protein aggregation from molecular structure is based on our developed AI-MD-Molecular surface curvature modelling platform, schematically presented in Fig. 1 (for methodology see also Methods). The modelling platform starts from the amino acid sequence of a given monoclonal antibody. Using this sequence, we then use AlphaFold^13^ to construct the 3D structure of the variable fragment (Fv) for each monoclonal antibody (note that in principle the approach could also be applied to other protein types). Next, to access the dynamics of proteins and the improved structure in the formulation, we used the AlphaFold-determined structure as input for molecular dynamics (MD) simulations (using the Gromacs package), generating a 100 ns trajectory for each antibody fragment. We then calculated selected surface features for each frame of the molecular dynamics simulation trajectory. Statistically significant surface features were identified and utilized to design machine learning models. We train our learning algorithms using leave-one-out cross-validation analysis on the experimentally measured dataset of 20 mAb aggregation rates (see Methods for details) reported in Ref.^35^. Specifically, this aggregation rate dataset is focused on high concentration mAb solutions as today used in biopharmaceutical formulations and is distinguished from the more prevalent low concentration datasets (e.g.^37^).

### Novel features based on molecular surface curvature

**Fig. 2:**
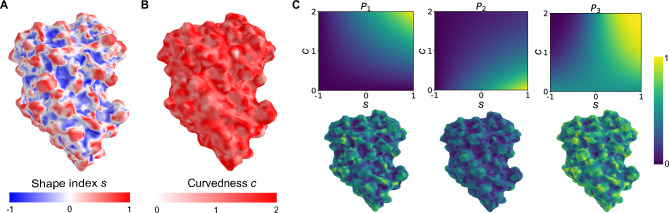
Local geometrical surface curvature for protein stability analysis. (**A**) Shape index - *s* and (**B**) curvedness - *c*, visualized for a test mAb. (**C**) Heat maps of penalty functions $P_1$, $P_2$ and $P_3$, for different values of *s* and *c* (top), and these same penalty functions visualized on a specific tested mAb (bottom).

We introduce a new protein surface feature, based on the physico-chemical and geometric aspects of the molecular surface, designed to evaluate aggregation propensity. Initially, we establish an equidistant mesh of points on the solvent-accessible surface. For each of these points, we compute the electrostatic potential and a smoothed projection of atom hydrophobicities, as explained in Methods, outlining the surface’s physico-chemical profile. These are further divided into separate positive and negative contributions. We then consider how the relative orientation and accessibility of these points on the interaction surface affect the protein association in a solution. For instance, if a point resides in a highly concave region of a protein, its interaction with an associating protein would be minimal, despite its solvent accessibility. To capture this, we calculate the principal curvatures at each point and employ the framework by Koenderick and Doorn^38^ (depicted in Fig. 2), utilizing the concept of shape index (*s*) and curvedness (*c*) (see Methods for details). Specifically, the shape index characterizes the overall shape of the surface around a point, distinguishing between protrusions, hyperbolic saddles, or cavities, whereas the curvedness gauges the intensity of that particular shape in each point.

To define a feature that also accounts for local surface shape, on all surface points, we introduce three distinct phenomenologically motivated penalty functions ($P_1, P_2, P_3$):

1a $$\begin{aligned} P_1= & \frac{s+1}{2} \cdot c, \end{aligned}$$

1b $$\begin{aligned} P_2= & \frac{s+1}{2} \cdot e^{-c}, \end{aligned}$$

1c $$\begin{aligned} P_3= & \text {erf}(s \cdot c), \end{aligned}$$

each corresponding to a distinct protein-protein interaction regime (Fig. 2).

Penalty function $P_1$ assumes that highly curved areas are more likely to be involved in protein-protein interactions, highlighting the accessibility of these regions. For instance, an atom situated at the end of a long exposed amino acid side-chain, such as Arginine, is prone to contact and interact with other proteins in a solution due to its high curvedness, and, notably, the positive charge of $C_{\zeta }$ atom at physiological pH.

Penalty function $P_2$ assumes that flatter, but still outward curved regions, are also prone to interaction, offering a more energetically favorable fit. $P_2$ assumes that protrusions with high curvedness may come into contact but at the cost of the surrounding atoms which implies a less complementary shape with the surface of other proteins, relying primarily on the strength of the most exposed atom. Differently, flatter regions are more likely to exhibit a cumulative effect of containing atoms, resulting in a stronger interaction.

Penalty function $P_3$ employs a sigmoid-type function, assuming that positively and outward curved regions, showing a plateau with increasing *s* and *c*, are more likely to be involved in protein-protein interactions than negatively curved regions. Similar to $P_2$, $P_3$ highlights the importance of a somewhat flat region, with similar penalization for both positive and negative shape index *s* at $c=0$. For protrusions, a non-linear rise in the penalization occurs with $P_3$, reaching a plateau value.

For each surface point, we explore three distinct cut-offs, corresponding to effective surface patch sizes, over which the average shape index and curvedness are computed. We consider values of 1 Å, 5 Å and 10 Å, where 1 Å captures the effect of about one atom, 5 Å describes the impact of a single amino acid, while 10 Å describes that amino acid and its closest neighboring amino acids.

Features are calculated from the evolution of the protein structure in time, as captured by MD simulations. Specifically, when calculating a quantity, we use its average value, i.e. the ensemble or time average of the instantaneous value across all potential states. Also, we consider the maximum, minimum, and the overall variance of the considered quantity, as we later show also determines whether a protein is likely to aggregate, capturing the reaction limiting effects of extreme states.

**Fig. 3:**
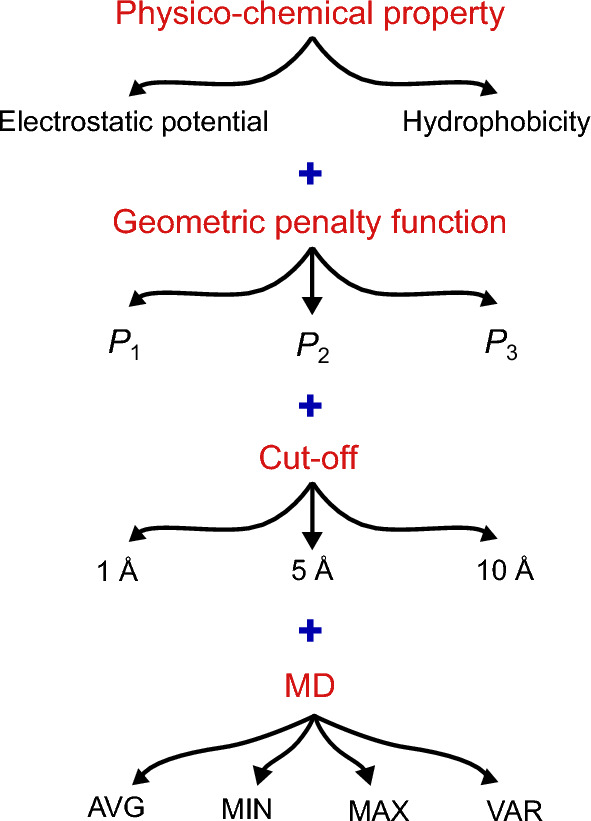
Construction of features for prediction of aggregation. The constructed features are all possible combinations of the indicated variables, distinctly including also surface curvature: physico-chemical property, geometric penalty function, cut-off and MD.

**Fig. 4:**
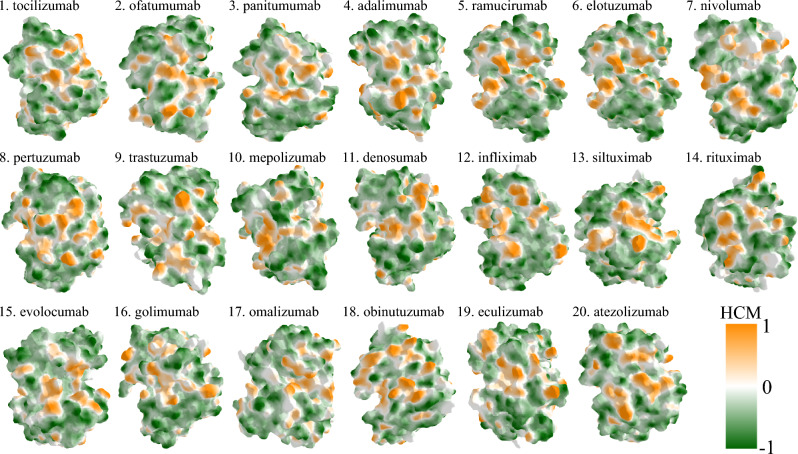
Spatial visualization of a selected feature for aggregation prediction. Specifically, we show LogP hydrophobicity combined with $P_3$ penalty function (selected from the best performing ML model in Table 1), visualized for all considered monoclonal antibodies.

Finally, we define the distinct feature *F* as a combination of all described factors:

2 $$\begin{aligned} F = \left\langle \, \sum _{A} \phi \cdot P(\text {cut-off}) \, \right\rangle _{\text {MD}}, \end{aligned}$$

where *A* is the protein region we calculate the feature over, such as complementarity-determining region (CDR) and Fv, $\phi$ is the physico-chemical property (such as hydrophobicity and electrostatic potential) and *P* is the penalty function ($P_1, P_2, P_3$) at a given cut-off. The overview of constructing the features is outlined in Fig. 3. Given that the Fv region is the most variable part in our dataset samples, we compute the features independently for the entire Fv region and each of the CDRs (L1, L2, L3 in the light chain and H1, H2, H3 in the heavy chain), as well as the whole CDR region. Fv region with one of the features is visualized for all mAbs in Fig. 4, where we name the curvature-dependent features ECM (Electrostatic Curvature Map) and HCM (Hydrophobic Curvature Map), respectively.

**Table 1:** Aggregation prediction performance. Performance of considered ML models, with the achieved *r*, $$\hbox{$\mathit{R}^2$}$$, MSE values and the corresponding three feature combinations. Note the excellent *r*$$=0.91$$ prediction with linear regression model. The following ML models are considered: Linear regression (Linear), K-nearest neighbours (KNN), Support vector machine (SVM), Gradient boosting (GRB) and Random forest (RF). Each feature is written as (see also Methods): physico-chemical property (HCM, ECM), hydrophobicity amino acid scale (logP, ww), signature of the feature (+, -), geometric penalty function ($$P_1$$, $$P_2$$, $$P_3$$), cut-off length (1 Å ...), MD time-dependency (MIN, MAX, AVG, VAR) and protein region of calculation (CDRH1-3, CDRL1-3, Fv).

Model	*r*	$$\hbox{$\mathit{R}^2$}$$	MSE	Feature combination
Linear	0.91	0.82	0.03	HCM (logP) +, $P_3$, 10 Å, AVG, CDRH1, HCM (logP) +, $P_2$, 5 Å, MAX, CDRH1, HCM (logP) +, $P_3$, 1 Å, MIN, CDRH1
KNN	0.89	0.31	0.10	HCM (ww) +, $P_2$, 1 Å, AVG, CDRL3, HCM (ww) +, $P_2$, 10 Å, MIN, CDRL1, HCM (logP) +, $P_2$, 10 Å, MIN, CDRH1
SVM	0.84	0.54	0.07	HCM (ww) +, $P_1$, 1 Å, AVG, CDRL3, HCM (ww) +, $P_3$, 5 Å, AVG, CDRL3, ECM +, $P_1$, 10 Å, VAR, CDRH2
GRB	0.79	0.49	0.07	HCM (logP) +, $P_2$, 10 Å, AVG, CDRH1, HCM (logP) +, $P_2$, 5 Å, MAX, CDRH1, HCM (ww) +, $P_3$, 5,Å, MIN, CDRL3
RF	0.78	0.45	0.08	HCM (ww) +, $P_3$, 1 Å, MAX, CDRL3, HCM (logP) +, $P_2$, 1 Å, MIN, CDRH1, HCM (logP) +, $P_2$, 5 Å, MIN, CDRH1

### Prediction of aggregation rate

We apply regression-based machine learning models on the computed final features *F* to predict the aggregation rate for 20 monoclonal antibodies in the considered database. We consider only features that correlate strongly with the aggregation rate (Pearson’s *r* $>0.4$; c. $\simeq 40$ best features are identified). We train the machine learning models on all possible combinations of three features, which proves to improve the accuracy of prediction in comparison to using just one or two features. To verify the approach we use leave-one-out cross-validation (LOOCV) to assure no overfitting occurs (see Methods).

**Fig. 5:**
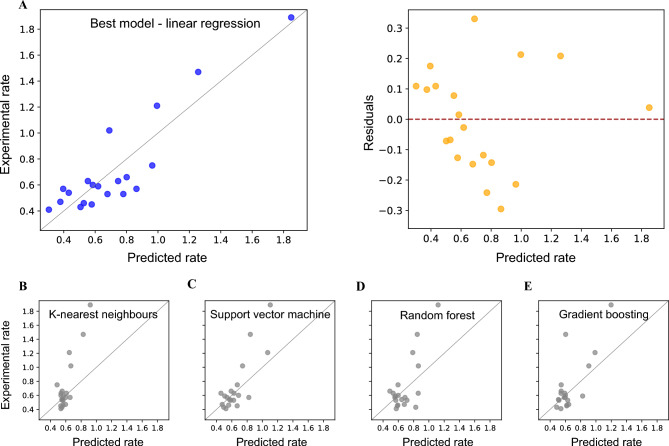
Prediction of monoclonal antibodies aggregation with our AI-MD-Molecular surface curvature modelling platform. Experimental vs predicted aggregation rates for: (**A**) best performing ML model - Linear regression and the corresponding residuals plot, (**B**) K-nearest neighbours, (**C**) Support vector machine, (**D**) Random forest and (**E**) Gradient boosting. All aggregation rates are given in units of ml/mg per week. Residuals in (**A**) are calculated as difference between experimental and predicted aggregation rate.

The best performing machine learning models according to Pearson’s correlation factor *r* are shown in Fig. 5 and listed in Table 1. The highest correlation factor is $r=0.91$, which to the best of our knowledge is better than any existing state-of-the-art^35,36^. The highest coefficient of determination $R^2=0.82$, and lowest mean squared error $\text {MSE}=0.03$ are achieved by using a linear regression model based on three features. All three features are based on the positive part of the hydrophobicity (MLP, see Methods) with atomic based Wildman-Crippen scale. Feature 1 includes $P_3$ penalty function, 10 Å cut-off, and is computed as a MD time average on the CDRH1 region. Feature 2 includes $P_2$ penalty function, 5 Å cut-off, and is computed on the CDRH1 region as a maximum value during the MD simulation. Feature 3 includes $P_3$ penalty function, 1 Å cut-off, and is computed on the CDRH1 region as a minimum value during the MD simulation. Other feature combinations and regression models also achieve correlation values above 0.78 (Table 1) with notably the best feature combinations including different hydrophobicity based metrics and only one feature in Table 1 describing the electrostatic potential. Visual inspection of Fig. 5 suggests that four data points with high experimental aggregation rates deviate significantly from the mean. To assess the robustness of our results, we recalculated the relevant performance metrics after excluding these points and observed an equivalent relative ranking of the models.

**Fig. 6:**
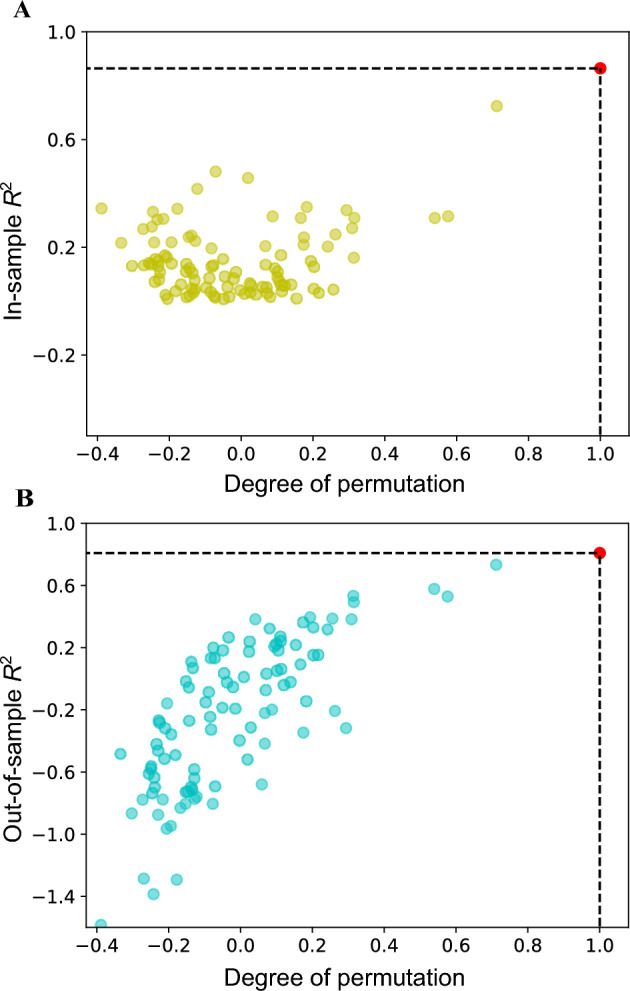
Permutation analysis of aggregation rate prediction. (**A**) In-sample and (**B**) out-of-sample coefficients of determination - $$\hbox{$\mathit{R}^2$}$$ for linear regression models are plotted for different permuted vector correlations. We use the three features combination from the best performing linear regression model in Table 1. Degree of permutation equal to 1 corresponds to the original experimental data, shown in red, while values approaching 0 or becoming negative indicate increasing levels of randomization (see Methods).

Permutation analysis is performed to show that overfitting in our ML model performance is minimal (see Table 2). Here we randomly permute the order of aggregation rates for all antibodies and see how the models perform after training. As the degree of permutation (correlation between the original and permuted aggregation rate vector) decreases, goodness-of-fit should decrease as well, and indeed, as shown in Fig. 6, the models perform consistently worse for lower degrees of permutation compared to the high ones. The calculated metrics for both the entire dataset (in-sample) and the leave-one-out cross-validation (LOOCV) dataset (out-of-sample) are given in Table 2. Finally, these results confirm that our approach avoids overfitting and appropriately captures a meaningful amount of variation in the original dataset, also in line with the PCA analysis (see Supplementary Fig. S1).

**Table 2:** Prediction performance (Pearson’s *r*, $$\hbox{$\mathit{R}^2$}$$ and MSE) on the complete and LOOCV dataset. The minimal decrease in out-of-sample performance observed with the LOOCV dataset indicates robust model generalization and good predictive power.

Data	*r*	$$\hbox{$\mathit{R}^2$}$$	MSE
All	0.93	0.86	0.02
LOOCV	0.91	0.82	0.03

## Discussion

Use of protein structure surface features is emerging as a potent approach for designing targeted drugs with fewer side effects and improved stability. In this work, we present an experimentally informed prediction of the aggregation rate of monoclonal antibodies, with prediction accuracy of 91% for a dataset of 20 mAbs under standard platform formulation conditions. Especially, we demonstrate the importance of local protein surface curvature in the surface feature construction, which in combination with hydrophobicity we show strongly correlates with the aggregation rate. Using molecular dynamics modelling, following AlphaFold structure prediction, is also shown to be important for achieving high prediction accuracy by providing core structural dynamics information into the surface feature construction. The prediction performance is further validated with leave-one-out cross-validation.

More specifically, our analysis of the top-performing ML models (Table 1) reveals interesting insights into the effects influencing protein aggregation. While electrostatic potential, incorporated in our work by the ECM feature, has been previously associated with solution behavior, mutual orientation, and viscosity^24,39,40^, our findings suggest may have less direct role in the aggregation than initially thought. It is believed that the aggregation process is primarily driven by the partial or complete unfolding of certain protein states^41^, which subsequently leads to non-specific associations due to exposed hydrophobic regions. Although pH-induced charge imbalances can potentially destabilize protein structures, such occurrences are statistically uncommon under the formulation conditions present in our dataset. Our results indicate that hydrophobic interactions, quantified through the HCM feature, play a more significant role in protein-protein interactions and subsequent aggregation. To further corroborate this, we performed ridge regression (L2 regularization) using the full set of input features. The resulting model highlighted the same key contributors (see Supplementary Fig. S2), all of which are either hydrophobicity based or related to surface area properties. Together, this suggests that the close proximity of two proteins, primarily mediated by hydrophobic regions, is -for the studied protein formulations- a key factor in initiating the destabilization process.

We have observed that the best machine learning algorithm we used was linear regression, as compared to other more complex algorithms (Table 1). In the training process, we use composite features that primarily describe charge and hydrophobicity, both effects which significantly influence a protein’s local geometry. For example, highly hydrophobic regions on a protein’s surface tend to avoid water, often burying themselves within the protein’s interior while still being partially exposed. These regions might not play a crucial role in protein-protein interactions, suggesting that an increase in surface hydrophobicity does not necessarily correlate linearly with a higher aggregation propensity. In contrast, our penalty functions account for this by selectively including only specific hydrophobic surfaces in the feature calculation. This selective approach enables us to detect a more accurate linear relationship between aggregation rates and spatially exposed hydrophobic surfaces, thereby enhancing the fit of our linear regression model.

The weak unfolding during our 100 ns MD simulation indicates we mainly describe native aggregation - the reversible association of native monomers^42^ which also aligns with the experimental temperatures ($45^{\circ }$C)^35^ and typical mAb melting ($T_{\text {m}}$) and aggregation onset ($T_{\text {agg}}$) temperatures, usually above $75^{\circ }$C^43^. While rare unfolding occurs at storage temperatures ($5^{\circ }$C), many aggregates can be reversible oligomers, supporting our algorithm’s predictions. Our conformational sampling is limited by computational constraints, as 100 ns mAb simulations take about a day on standard GPU clusters, while characteristic backbone fluctuations occur in microseconds^44^. Nevertheless, this proves to be a long enough time for the correlation between the relevant calculated features and the aggregation rate to converge and provide meaningful insight (see Supplementary Fig. S3). Future work could employ enhanced sampling techniques like metadynamics^45^ or parallel tempering^46^ to improve modeling.

Finally, this work is fully inline with the challenge of developing general in-silico aggregation prediction models of proteins, as of direct relevance for the industrial biopharmaceutical drug and formulation development processes. The ability to predict aggregation facilitates precise molecule design, enabling targeted drug development with reduced side effects^47^. In formulation development, it also guides the creation of stable formulations with optimized delivery systems, extending shelf life and allowing for higher drug concentrations at low viscosity. More generally, the ability to predict protein aggregation with high precision contributes to the patient-friendly administration of biopharmaceutical drugs by reducing injection volumes, enhancing convenience, and supporting home use^48^.

## Methods

### Structure calculation with AlphaFold and molecular dynamics

The amino acid sequences of the 20 monoclonal antibodies were used to construct the 3D structure of the Fv region of each antibody using AlphaFold^13^. The obtained Fv structure was used as the inintial condition for molecular dynamics simulation, which was performed using GROMACS molecular dynamics package^49^ with the OPLS-AA force field^50^. The antibody fragment was placed in a simulation box extending at least 2 nm beyond the antibody in all directions and the box was filled with explicit solvent molecules using the SPC/E water model^51^. The system pH was set at 6.0 using PROPKA3^52^ and ions were added to the simulation box to ensure a neutral environment. Energy minimization of the system was done with the steepest descent algorithm. The system was first equilibrated through successive NVT and NPT ensemble simulation runs for a joint duration of 10 ns. This was followed by a 100 ns production run which was performed in the NPT ensemble at 298 K and the pressure of 1 bar. The integration time step was set at 2 fs, resulting in 5000 frames for each antibody fragment.

### Surface features

We calculate the introduced novel surface features using an in-house developed scrypt in Python 3.10. The electrostatic potential on the surface is calculated with Delphi software^53^, using the OPLS-AA forcefield, which provides a framework for solving the Poisson-Boltzmann equation

3a $$\begin{aligned}&- \nabla \cdot \epsilon \nabla \phi ({\textbf {r}}) = \sum _i c_i q_i e^{-q_i \phi / k_B T} + \rho ({\varvec{r}}), \end{aligned}$$

3b $$\begin{aligned}&\rho ({\textbf {r}}) = \sum _j Q_j \delta ({\varvec{r}}-\varvec{r_j}), \end{aligned}$$

where $\epsilon$ is the dielectric constant (80 for bulk water and 4 for protein interiors^54^, $\phi$ the electrostatic potential, $k_B$ the Boltzmann constant, *T* the temperature and $\rho ({\textbf {r}})$ the spatial charge density. $c_i$ and $q_i$ denote the concentration and charge of ions, respectively, and $Q_j$ represents the charge of protein atoms.

We project normalized atom hydrophobicities onto the surface points using the concept of Molecular Lipophilicity Potential (MLP)^55^, defined as

4a $$\begin{aligned}&MLP = \sum _i p_i f_i, \end{aligned}$$

4b $$\begin{aligned}&p_i = \frac{g(d_i)}{\sum _j g(d_j)}, \end{aligned}$$

4c $$\begin{aligned}&g(d) = \frac{1}{e^{1.5(d-4.0)}+1} \end{aligned}$$

where MLP is the sum of nearby atoms’ normalized hydrophobicities $f_i$, with $p_i$ representing the weights of the contributions and *g* being a Fermi type distance function, with parameters defined by the range of the hydrophobic effect. We assign the hydrophobicity using two top contending hydrophobicity scales, based on their correlation to HIC (hydrophobic interaction chromatography) measurements^56^. These are the amino acid based Wimley-White scale^57^ and the atomic based Wildman-Crippen scale^58^.

The definition and triangulation of surface points are performed using MSMS software^59^. For each point, we extract coordinates, normal vector components, and information about nearest neighbors based on triangulation. Principal curvatures $\kappa _1$ and $\kappa _2$ at each point are then computed using the algorithm proposed by Hamann^60^. Subsequently, shape index (*s*) and curvedness (*c*)^38^ are calculated using these principal curvatures as

5a $$\begin{aligned}&s = \frac{2}{\pi } \arctan \frac{\kappa _2+\kappa _1}{\kappa _2-\kappa _1} \quad (\kappa _1 \ge \kappa _2), \end{aligned}$$

5b $$\begin{aligned}&c = \sqrt{\frac{\kappa _1^2 + \kappa _2^2}{2}}, \end{aligned}$$

and averaged over nearby surface points, employing different cutoffs based on Euclidean distance. The whole feature building procedure is shown in Fig. 3.

Also, we calculate different known classification features used in literature^35,36^ to allow for comparison. For example, our implementation of SASA involves evenly distributing points on the surface using the Fibonacci sphere and counting solvent-accessible points, according to the Shrake-Rupley (“rolling probe”) algorithm^61^. Partial charges for SCM calculation are assigned and extracted through GROMACS as part of the MD simulation process. In our SAP implementation, we use the Wimley-White and the Wildman-Crippen scale, same as mentioned earlier.

### Dataset of relevant mAbs and aggregation rates

We train our learning algorithms on the experimentally measured dataset of 20 mAb aggregation rates, as selected also in Ref.^35^ (Bevacizumab was excluded due to its aggregation rate value beyond 3 standard deviations from the mean and therefore outside the data behaviour ML models can effectively capture^62^). The aggregation rate was measured at pharmaceutically relevant high mAb concentration of $\text {c} = 150\,\text {mg/mL}$ at $40^\circ \text {C}$ and at pH 6.0. Out of the 20 mAbs considered, 4 show aggregation rate higher than $0.8\cdot 10^{-4}\,\frac{\text {mL}}{\text {mg week}}$, which makes them less suitable for long-term storage and distribution typically required in biopharmaceutical applications. Each of the considered mAbs is FDA approved and has a published amino acid sequence.

### Machine learning prediction of aggregation rate

The aggregation rate dataset is used to train the algorithm and validate its results. We test several regression models including Linear regression, K-nearest neighbours, Support vector machine, Random forest and Gradient boosting. Note, that Neural networks are not used due to a small size of the available dataset.

We employ the leave-one-out cross-validation (LOOCV) technique which enables us to systematically test and identify the most effective model. Note, that LOOCV approach addresses dataset imbalances more effectively than the conventional train-test split, where certain classes may be underrepresented in the training set, impacting optimal predictions in the test set. LOOCV also allows the entire dataset to be used as the test set.

To streamline our analysis, we initially narrow down the feature space by selecting only those features exhibiting a significant correlation with the aggregation rate (Pearson’s *r* $$\ge$$ 0.4 and *p*-value < 0.05). As an additional filter, we exclude all features where values for any of the mAbs deviate by more than 3 standard deviations from the mean. Subsequently, we explore all possible three feature combinations, training separate models for each combination. The top combinations are determined based on their correlation between predicted and experimental aggregation rates (Pearson’s *r*), coefficient of determination ($$\hbox{$\mathit{R}^2$}$$), and mean square error (MSE).

In permutation analysis we take the experimental aggregation rate vector of all 20 monoclonal antibodies and generate 100 aggregation rate vectors with randomly permuted values. The degree of permutation is defined as the correlation coefficient (Pearson’s *r*) between the original and the permuted aggregation rate vector. For each of the generated aggregation rate vectors, we train the model independently using the same features as with the unpermuted aggregation rate vector. We then record the in-sample coefficient of determination ($$\hbox{$\mathit{R}^2$}$$), calculated using a model trained on the full dataset, as well as the out-of-sample $$\hbox{$\mathit{R}^2$}$$, obtained via LOOCV by evaluating the model on the held-out and predicted values. This allows us to assess both the fit and generalization accuracy of the model.

## Supplementary Information

Supplementary Information.

## Data Availability

Data are available from the authors upon reasonable request.
