# Supplementary material for: Prediction of aggregation in monoclonal antibodies from molecular surface curvature
Source: Sci Rep. 2025 Aug 2;15:28266. doi: 10.1038/s41598-025-13527-w (PMC12317995; doi:10.1038/s41598-025-13527-w)
Supplement: Supplementary file 1 — Supplementary Information. [file 41598_2025_13527_MOESM1_ESM.pdf]

Supplementary Information for Prediction of aggregation in monoclonal antibodies from molecular surface curvature

Benjamin Knez,[1,2] Lara Erzin,[2] Žiga Kos,[2,3,4] Drago Kuzman,[1] and Miha Ravnik *[2,4,3]

[1]*Novartis LLC, Verovškova 57, 1000 Ljubljana, Slovenia*
[2]*Faculty of Mathematics and Physics, University of Ljubljana, Jadranska 19, 1000 Ljubljana, Slovenia*
[3]*International Institute for Sustainability with Knotted Chiral Meta Matter (WPI-SKCM²), Hiroshima University, Higashi-Hiroshima, Japan*
[4]*Department of Condensed Matter Physics, Jožef Stefan Institute, Ljubljana, Slovenia*

PCA ANALYSIS OF MONOCLONAL ANTIBODY FEATURE SPACE

In order to unveil any inherent patterns or classes within the data, we conduct principal component analysis (PCA) using all features (Fig. S1).

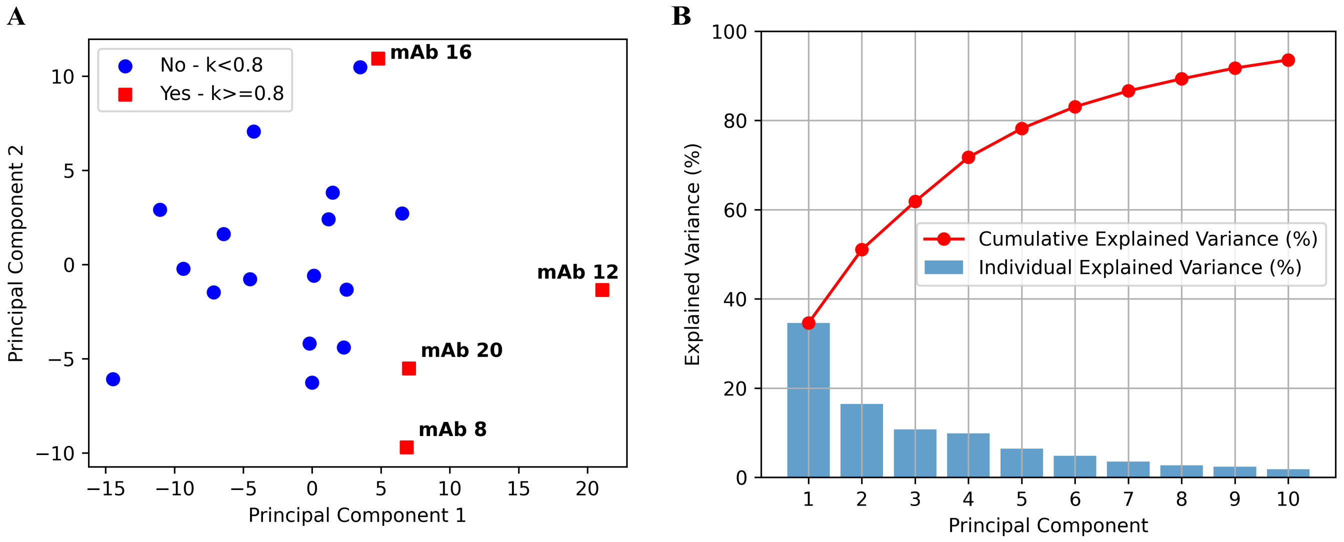

FIG. S1. **Principal Component Analysis (PCA) of calculated antibody surface features.** (A) Data points represent individual antibodies plotted against the first two principal component axes, which capture the maximum variance in the dataset (encapsulating 52% of the information). Colors indicate aggregation rate categories: high (red, $k \geq 0.8$ ml/mg per week) and low (blue, $k < 0.8$ ml/mg per week). (B) Pareto plot showing the explained variance of the top 10 principal components. Bars represent the individual variance explained by each component, and the red line indicates the cumulative variance.

The color coding scheme distinguishes between antibodies with high aggregation rates (red, $k \geq 0.8$ ml/mg per week) and those with low aggregation rates (blue, $k < 0.8$ ml/mg per week). This visualization reveals a pattern: antibodies prone to high aggregation rates predominantly cluster together, forming a relatively well-defined group in the PCA space (notably, mAb sample number 16 - rituximab deviates from this pattern). This clustering suggests a common set of underlying physicochemical properties captured in the calculated structural features that may predispose these antibodies to increased aggregation.

RIDGE REGRESSION (L2 REGULARIZATION)

We additionally built a ridge regression model, selecting the optimal regularization parameter alpha (α) by maximizing predictive accuracy through 5-fold cross-validation.

As shown in Figure S2, the features with the highest weights are predominantly related to hydrophobicity (such as HCM and SAP) and solvent-accessible surface area (SASA). These findings further support our conclusion that hydrophobic surface regions are central to driving protein-protein interactions and aggregation in the studied formulations.

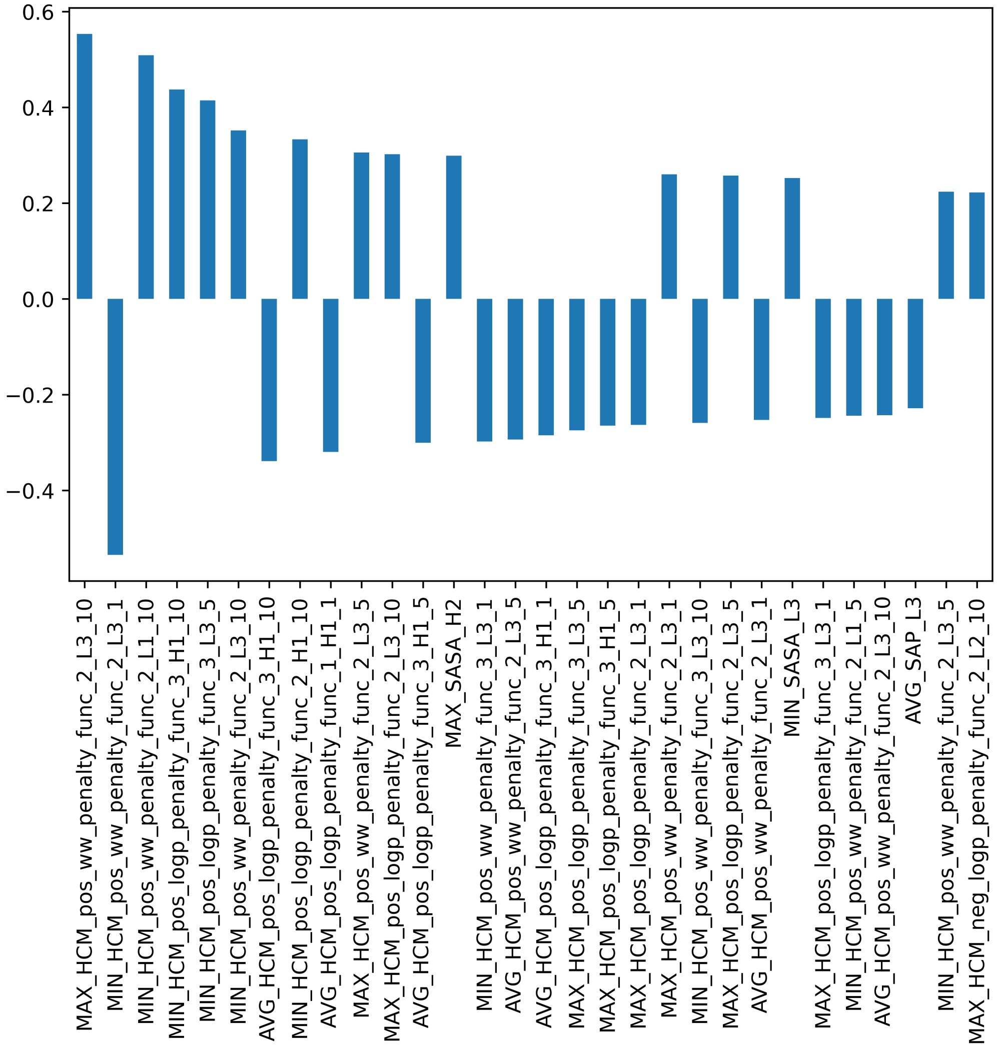

FIG. S2. **Top 30 feature coefficients from ridge regression.** Shown are the 30 features with the largest absolute coefficients (weights) in the ridge regression model, using an optimal $\alpha = 0.0001$. Features are ordered by the magnitude of their contribution to the model.

FEATURE EVOLUTION THROUGH TIME

We have analyzed the temporal evolution of correlation between selected features and the aggregation rate, captured through MD simulations.

Figure S3 reveals a non-random pattern in the correlation's evolution. Notably, the correlation converges to a stable value over the course of a 100 ns production MD run. This convergence suggests two important insights. First, a meaningful relationship exists between the selected features and the aggregation rate, reinforcing the validity and reliability of our predictive model. Second, the 100 ns simulation duration is sufficient to capture critical aspects of protein dynamics relevant to aggregation behavior, providing valuable information about the necessary timescales for dynamics description.

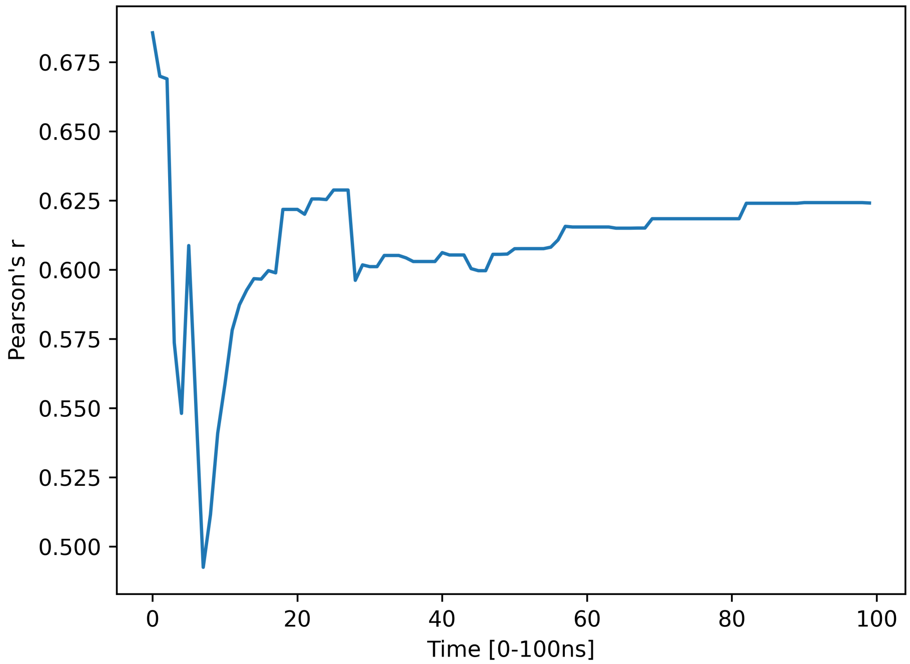

FIG. S3. **Temporal evolution of correlation between a key feature and aggregation rate.** The feature (HCM (logP) +, P_3, 10 Å, AVG, CDRH1) is calculated at each time step, including the part of the simulation that has already passed. The plot shows the progression of the correlation coefficient between this feature and the aggregation rate over the course of the simulation.
